# Socioeconomic background in relation to stage at diagnosis, treatment and survival in women with breast cancer

**DOI:** 10.1038/sj.bjc.6603622

**Published:** 2007-02-20

**Authors:** A Downing, K Prakash, M S Gilthorpe, J S Mikeljevic, D Forman

**Affiliations:** 1 Cancer Epidemiology Group, Centre for Epidemiology & Biostatistics, University of Leeds, 30-32 Hyde Terrace, Leeds, LS2 9LN, UK; 2 Cancer Research UK Clinical Centre, St James' Hospital, Beckett Street, Leeds LS9 7TF, UK; 3 Leeds General Infirmary, Great George Street, Leeds LS1 3EX, UK; 4 Biostatistics Unit, Centre for Epidemiology & Biostatistics, University of Leeds, 30-32 Hyde Terrace, Leeds LS2 9LN, UK; 5 St James' Hospital, Beckett Street, Leeds LS9 7TF, UK; 6 Northern & Yorkshire Cancer Registry and Information Service, Arthington House, Cookridge Hospital, Leeds, LS16 6QB, UK

**Keywords:** breast cancer, socioeconomic background, stage at diagnosis, treatment, survival

## Abstract

In a large population-based series of invasive breast cancer patients, we investigated socioeconomic background (SEB) in relation to (a) stage at diagnosis; (b) treatment pattern; and (c) 5-year survival. Women diagnosed during 1998–2000 and resident in the Northern and Yorkshire regions of England were identified from the cancer registry database (*N*=12 768). Logistic regression and Cox proportional hazards analyses were used to estimate associations between SEB (defined using the Townsend Index for area of residence) and tumour stage, treatment pattern, and survival. Living in a more deprived area was associated with increased likelihood of being diagnosed with stage III or IV disease (age-adjusted odds ratio (OR) 1.13; 95% confidence interval (CI) 1.08–1.18 per quartile increase in Townsend score), and, after adjustment for age and stage, reduced odds of having surgery (OR 0.85; 95% CI 0.80–0.91), and receiving radiotherapy (OR 0.91; 95% CI 0.88–0.94). Amongst patients receiving surgery, those living in more deprived areas had decreased odds of having breast conserving surgery (age plus stage-adjusted OR 0.92; 95% CI 0.89–0.95). Living in a more deprived area was also associated with increased mortality (age- plus stage-adjusted hazard ratio 1.08; 95% CI 1.05–1.11). These effects may operate through several pathways, such as later presentation leading to advanced disease.

Breast cancer is the commonest female cancer in the UK, accounting for about 30% of all cancers in women (Cancer Research UK, 2006). Owing to a combination of its natural history, methods for early diagnosis and effective treatment, survival from breast cancer is better than that of many other cancers (Department of Health, 2000). Differences in survival according to socioeconomic background (SEB) have, however, been reported from the UK, with women from more deprived areas showing lower survival rates than those from more affluent areas of residence, with differences of 6% at 5 years (Coleman *et al*, 2004), and 10% at 10 years (Thomson *et al*, 2001).

Socioeconomic background has been associated with differences in tumour biology, such as type, grade, and oestrogen receptor (ER) status (Thomson *et al*, 2001; Taylor and Cheng, 2003), stage at diagnosis (Macleod *et al*, 2000a; Adams *et al*, 2004), variation in diagnostic investigations and treatments (Twelves *et al*, 1998) and comorbidity (Macleod *et al*, 2000b). However, certain findings have been inconsistent, such as differences in stage by level of area deprivation, which have been observed in some (Macleod *et al*, 2000a; Adams *et al*, 2004) but not in other studies (Carnon *et al*, 1994; Brewster *et al*, 2001; Thomson *et al*, 2001; Taylor and Cheng, 2003). Attempts to explain socioeconomic differences in survival on the basis of stage and treatment have also produced conflicting results, which may reflect differing health service provisions and socioeconomic structures in the relevant countries (Thomson *et al*, 2001).

We have investigated the relationship between SEB and (a) stage at diagnosis, (b) treatment patterns and (c) 5-year survival, in a large population-based sample of invasive breast cancer patients diagnosed in 1998 to 2000.

## MATERIALS AND METHODS

Women with invasive breast cancer (ICD10; (World Health Organisation, 1992; code C50) diagnosed in the years 1998–2000 and resident in the Northern and Yorkshire regions were identified from the Northern and Yorkshire Cancer Registry and Information Service database. Patient age and tumour stage at diagnosis (using the TNM system; Singletary *et al*, 2002), treatment (type of surgery and whether or not radiotherapy (RT), chemotherapy (CT), or hormone therapy (HT) were given), the time taken to receive a hospital appointment after GP referral (appointment delay), and the time taken to receive treatment after diagnosis (treatment delay) were extracted. SEB was defined at the 1991 enumeration district level of residence using the Townsend index (Townsend and Phillimore, 1988) and matched to the patient using their postcode. An area deprivation score could not be obtained in 40 cases.

All factors were examined in relation to quartiles of area deprivation (Townsend score) (Table 1). Logistic regression analyses were used to examine any associations between SEB and stage or treatment (type of surgery, use of adjuvant treatments, appointment delay and treatment delay), whereas Cox proportional hazards analyses (Cox, 1972) were used to evaluate any association between SEB and 5-year survival. Models were adjusted for age or age plus stage. Proportionality assumptions were verified for all survival models.

The Townsend score was scaled and included as a continuous variable such that the resulting odds ratios (ORs) or hazard ratios (HRs) represent a change in the outcome per quartile increase in the Townsend score. As details of some factors were not available for all patients (Table 1), analyses were conducted after the exclusion of ‘unknowns’ for the factor under investigation.

## RESULTS

A total of 12 768 patients were included in the analyses. A higher proportion of patients living in the most deprived quartile (45.3%; 95% confidence interval (CI) 43.6–47.0) were older than 65 years at diagnosis compared to those living in the most affluent quartile (35.9%; 95% CI 34.2–37.6) (Table 1). Patients living in the most affluent quartile were diagnosed more frequently with stage I disease (42.0%; 95% CI 40.2–43.8) than those in the most deprived quartile (36.3%; 95% CI 34.6–38.1), whereas a higher proportion of those with metastatic disease (stage IV) lived in the most deprived quartile (7.0%; 95% CI 6.1–8.0) compared to the most affluent quartile (3.9%; 95% CI 3.2–4.6). A higher proportion of patients living in the most affluent quartile had breast-conserving surgery (BCS) compared to those in the most deprived quartile, either alone (40.4%; 95% CI 38.7–42.1 *vs* 31.7%; 95% CI 30.1–33.3) or before mastectomy (10.4%; 95% CI 9.4–11.5 *vs* 7.4%; 95% CI 6.4–8.3), a greater proportion of those living in the most deprived quartile received no surgery (22.2%; 95% CI 20.7–23.6 *vs* 13.2% ; 95% CI 12.0–14.4). A higher proportion of the patients resident in the most affluent quartile received RT (58.3%; 95% CI 56.6–60.0) compared to those in the most deprived quartile (49.0%; 95% CI 47.3–50.7). There was no statistically significant difference in the proportion of patients having BCS with subsequent RT (86.3%; 95% CI 84.2–88.5 and 88.2%; 95% CI 86.4–90.0 in the most deprived and most affluent quartiles respectively) nor in the proportions receiving HT (82.1%; 95% CI 80.7–83.4 *vs* 80.4%; 95% CI 79.0–81.7) and CT (26.4%; 95% CI 24.9–28.0 *vs* 29.2%; 95% CI 27.6–30.8). Women living in the most affluent quartile were more frequently seen within 14 days of referral (62.7%; 95% CI 60.6–64.7) compared to those in the most deprived quartile (58.6%; 95% CI 56.6–60.6) but this difference was less for beginning treatment within 14 days of being diagnosed (61.9%; 95% CI 60.2–63.6 *vs* 59.3%; 95% CI 57.5–61.0).

The logistic regression analyses show that, after adjustment for age, living in a more deprived area was associated with a significantly increased odds of being diagnosed with stage III or IV disease (OR 1.13; 95% CI 1.08–1.18 per quartile increase in Townsend score) (Table 2). After adjustment for age and stage, living in a more deprived area was significantly associated with decreased odds of receiving surgery (OR 0.85; 95% CI 0.80–0.91) and RT (OR 0.91; 95% CI 0.88–0.94). Amongst patients receiving surgery, living in a more deprived area was associated with significantly decreased odds of having BCS (OR 0.92; 95% CI 0.89–0.95 per quartile increase), and significantly increased odds of having a mastectomy (OR 1.08; 95% CI 1.05–1.12). Area Townsend score was not significantly associated with the odds of CT (OR 0.97; 95% CI 0.93–1.01), although the association was of borderline significance for HT (OR 1.04; 95% CI 1.00–1.08). Living in a more deprived area was also associated with an appointment delay of more than 14 days (OR 1.07; 95% CI 1.03–1.11) and a treatment delay of more than 14 days (OR 1.05; 95% CI 1.02–1.08). The age plus stage-adjusted results differed little from the age-adjusted results, and only in the case of treatment delay did the estimate become statistically significant after additional adjustment for stage.

Following age adjustment, living in a more deprived area was associated with an increased risk of death (HR 1.11; 95% CI 1.08–1.13 per quartile increase in Townsend score), which was reduced after adjusting for stage (HR 1.08; 95% CI 1.05–1.11) (Table 3). When these analyses were repeated separately for patients undergoing BCS, having a mastectomy and having no surgery, there was a stronger association between Townsend score and survival for the BCS group (age plus stage-adjusted HR 1.13; 95% CI 1.07–1.19 per quartile increase) than for the mastectomy group (HR 1.04; 95% CI 1.00–1.08) and the no surgery group (HR 1.03; 95% CI 0.98–1.08); these results differed little from the age-adjusted results.

## DISCUSSION

Our results show that patients living in more deprived areas were more likely to be diagnosed with more advanced (stage III or IV) disease. This may be related to later presentation and lower rates of mammography screening in lower SEB areas. This finding contrasts with those of several other studies (Brewster *et al*, 2001; Thomson *et al*, 2001; Taylor and Cheng, 2003). However, this study was based on a much larger number of patients and staging data were available in over 90% of cases.

Women living in more deprived areas were less likely to have surgical treatment than those in affluent areas, and when they had surgery they were more likely to have a mastectomy. Adjustment for stage of disease at diagnosis made little difference to these results. Decisions about whether or not patients receive surgery and the type of surgery performed are the outcome of, in many cases, a complex interaction between stage and evaluation of the choices offered to patients by their clinical management team, the manner in which these choices are presented, and perceptions of the risks and benefits involved. The extent to which the latter factors may vary by SEB is poorly understood.

RT was the only adjuvant treatment found to be significantly related to SEB, with women in more deprived areas being less likely to receive this. RT for breast cancer can be intensive, involving visits to a RT centre for several days each week over several weeks, and this may have more impact on the treatment decisions of women in socially deprived areas. Other UK studies have found no difference in the uptake of RT by SEB (Macleod *et al*, 2000b; Thomson *et al*, 2001). When analysis was restricted to BCS patients, there was no significant difference in RT by SEB. BCS followed by RT is considered an optimal treatment and it is reassuring that there were no differences in this by SEB. Previous results on CT and HT have been inconclusive, with some studies reporting an association with SEB (Thomson *et al*, 2001; Taylor and Cheng, 2003) and others not (Macleod *et al*, 2000b; Taylor and Cheng, 2003). In our study, there was no association between Townsend score and CT, but there was a weak borderline significant association with HT. The decision to give adjuvant therapies is complex and depends upon such factors as whether the patient has undergone surgery and the ER status of the tumour, making it difficult to draw any firm conclusions from these results.

Current guidelines state that women should not wait longer than 14 days between GP referral and a first hospital appointment or between diagnosis and beginning treatment (National Institute for Clinical Excellence, 2002). During our study period, these targets were met in approximately 60% of cases. Women living in more deprived areas were more likely to wait more than 14 days for a hospital appointment than those in more affluent areas, though these results are limited by the fact that data were missing in 32% of cases. A weaker association between SEB and treatment delay was also apparent, which could indicate an influence of SEB on management. Nevertheless, although long delays by the patient are associated with decreased survival (Richards *et al*, 1999), delays by providers have not previously been linked to poorer outcomes (Sainsbury *et al*, 1999).

Living in a more deprived area was associated with lower 5-year survival. The age-adjusted HR was 1.11 (95% CI 1.08–1.13) per quartile increase in Townsend score and this reduced to 1.08 (95% CI 1.05–1.11) when additionally adjusted for stage. A previous study found that adjusting for stage did not substantially change the estimated HRs for deprivation category (Kaffashian *et al*, 2003). One explanation for this is that the adjustment for stage is insufficient because stage is not a sensitive enough measure to capture the variation in disease. For example, stage IV patients could present with early metastatic spread or may have such advanced disease that no treatment, other than palliative care, can be given. Stage therefore fails to act as an adequate proxy for preceding confounders associated with severity of disease.

Several others studies have concluded that prognostic factors, such as tumour stage, grade, size, and lymph node status only partly account for the differences in survival by SEB (Carnon *et al*, 1994; Macleod *et al*, 2000a; Brewster *et al*, 2001). Another factor associated with SEB is ER status, with patients living in more deprived areas having higher levels of ER negative tumours (Twelves *et al*, 1998; Thomson *et al*, 2001; Taylor and Cheng, 2003). It has been reported that these tumours appear to have a worse prognosis, though the difference in ER status was estimated to explain only about a third of the survival difference by SEB (Thomson *et al*, 2001).

As well as prognostic factors, there are many other potential confounders associated with SEB and with survival. Examples that are currently unavailable from cancer registry data include level of education and comorbidity. A higher level of education amongst those living in the more affluent areas may result in a higher degree of health awareness, better perception of symptoms and less delay in seeking medical care (Dalton *et al*, 2006), which may in turn lead to improved survival. The presence of co-morbidity has been associated with reduced survival in breast cancer (Houterman *et al*, 2004; Louwman *et al*, 2005), and patients from deprived areas may have a higher level of comorbidity (Macleod *et al*, 2000b).

It appears that the association between SEB and survival is stronger in patients undergoing BCS, which cannot be explained by RT, as there were no RT differences between deprived and affluent areas. However, the BCS group will contain a higher proportion of screen-detected tumours, which are likely to be smaller and have a good prognosis. If the number of screen-detected cases was higher in the more affluent areas, this may explain some of the observed survival advantage. Again, the staging information may not have been sensitive enough to capture such variation in disease.

There may also be some bias in these results. Potentially, many risk factors linked to SEB might affect breast cancer outcome. Statistical adjustment for a confounder or its proxy on the causal path from exposure to outcome may be subject to the bias known as the reversal paradox (Stigler, 1999; Kirkwood and Sterne, 2003). This has recently been shown to be a potentially serious problem (Tu *et al*, 2005; Hernandez-Diaz *et al*, 2006) and may explain some of the conflicting results concerning breast cancer outcomes by SEB. For example, a causal relationship may exist between SEB and stage, perhaps mediated through reduced health awareness and later presentation amongst those in more deprived areas. If stage lies on the causal path, then the adjustment for confounding becomes mixed with potential bias from the reversal paradox. In this case, the age-adjusted estimates, though unbiased, would represent the confounder-unadjusted results, and the age- plus stage-adjusted estimates would represent the (biased) confounder-adjusted results. It then becomes impossible to determine a confounder-adjusted unbiased estimate of the impact of SEB on survival. Despite these complexities, it is likely that the true public health effect of SEB on survival is that of the age-adjusted estimate.

Alternatively, one might make use of stage lying on the causal path, as it is then a candidate for an intermediate end point (Freedman *et al*, 1992), that is, a marker or event that is assessed before the outcome (survival in this case), and sufficiently associated with the outcome that it acts as its proxy. From this perspective, the age- plus stage-adjusted model yields estimates of the joint effect of SEB and stage on survival. Using Freedman's method, it can be estimated how much of the SEB effect on survival is mediated via the influence of SEB on stage (26% using the age-adjusted estimate as the denominator). This figure suggests that stage is not an ideal choice of intermediate end point, though this might be in part owing to measurement error and missing data associated with stage. Whether stage is considered as a proxy for preceding confounders and/or an intermediate end point, it seems poor in both respects.

This study used an area-based measure of SEB, as an individual measure is not available from cancer registry data. Owing to the ecological fallacy (Robinson, 1950), these results cannot be extended to individual women living in each area. We are exploring alternative methodologies to address both the ecological problem and potentially biased adjustment for confounders that lie on the causal path between SEB and survival.

This study has shown that breast cancer patients living in more deprived areas were more likely to be diagnosed with later stage disease than those in more affluent areas and that there were significant differences in the type of treatment received. Living in a more deprived area was associated with decreased survival, perhaps owing to later presentation and more complex treatment decisions. Part of this survival gap may be reduced through policies to encourage earlier diagnosis and appropriate treatment uptake, but the association between SEB and other factors, such as comorbidity, requires further investigation.

## Figures and Tables

**Table 1:** Distribution of patient, tumour, and treatment factors in the study population, by deprivation category

	**All patients**	**Townsend quartile**			
**Variable**	***N* (%)**	**I (affluent)**	**II**	**III**	**IV (deprived)**
*Age*					
<50	2698 (21.1)	696 (21.8)	706 (22.2)	657 (20.6)	639 (20.0)
50–64	4812 (37.7)	1354 (42.3)	1254 (39.3)	1097 (34.4)	1107 (34.7)
65+	5258 (41.2)	1148 (35.9)	1227 (38.5)	1438 (45.1)	1445 (45.3)
					
*Stage*					
I	4539 (39.2)	1235 (42.0)	1202 (40.8)	1063 (37.3)	1039 (36.3)
II	5576 (48.1)	1380 (47.0)	1414 (48.0)	1416 (49.7)	1366 (47.8)
III	900 (7.8)	210 (7.1)	220 (7.5)	217 (7.6)	253 (8.8)
IV	578 (5.0)	114 (3.9)	110 (3.7)	153 (5.4)	201 (7.0)
					
*Surgery*					
BCS alone	4605 (36.2)	1288 (40.4)	1250 (39.4)	1058 (33.3)	1009 (31.7)
Mastectomy alone	4721 (37.1)	1146 (36.0)	1141 (35.9)	1201 (37.8)	1233 (38.8)
BCS and mastectomy^a^	1178 (9.3)	332 (10.4)	319 (10.1)	293 (9.2)	234 (7.4)
No surgery	2217 (17.4)	421 (13.2)	464 (14.6)	627 (19.7)	705 (22.2)
					
*Radiotherapy*					
Yes	6734 (53.6)	1831 (58.3)	1755 (56.1)	1602 (50.9)	1546 (49.0)
No	5837 (46.4)	1309 (41.7)	1371 (43.9)	1548 (49.1)	1609 (51.0)
					
*Surgery and RT* b					
BCS alone	3951 (86.6)	1123 (88.2)	1068 (86.5)	894 (85.1)	866 (86.3)
Mastectomy alone	2079 (44.8)	524 (46.8)	517 (46.3)	525 (44.4)	513 (42.2)
BCS and mastectomy^a^	396 (34.3)	120 (37.5)	109 (34.9)	100 (35.4)	67 (28.9)
No surgery	304 (13.7)	62 (14.8)	61 (13.2)	81 (12.9)	100 (14.2)
					
*Hormone therapy*					
Yes	10285 (81.2)	2540 (80.4)	2535 (80.4)	2605 (82.1)	2605 (82.1)
No	2376 (18.8)	621 (19.6)	618 (19.6)	568 (17.9)	569 (17.9)
					
*Chemotherapy*					
Yes	3536 (28.1)	917 (29.2)	898 (28.7)	888 (28.2)	833 (26.4)
No	9036 (71.9)	2223 (70.8)	2231 (71.3)	2262 (71.8)	2320 (73.6)
					
*Appointment delay*					
0–14 days	5290 (60.7)	1302 (62.7)	1258 (60.1)	1397 (61.4)	1333 (58.6)
15–28 days	2051 (23.5)	483 (23.2)	489 (23.4)	516 (22.7)	563 (24.8)
29–60 days	1028 (11.8)	219 (10.5)	250 (12.0)	273 (12.0)	286 (12.6)
61–180 days	351 (4.0)	74 (3.6)	95 (4.5)	90 (4.0)	92 (4.0)
					
*Treatment delay*					
0–14 days	7461 (60.2)	1932 (61.9)	1845 (59.6)	1855 (60.1)	1829 (59.3)
15–28 days	3442 (27.8)	842 (27.0)	881 (28.5)	844 (27.3)	875 (28.4)
29–60 days	1342 (10.8)	317 (10.2)	335 (10.8)	350 (11.3)	340 (11.0)
61–180 days	141 (1.1)	28 (0.9)	33 (1.1)	38 (1.2)	42 (1.4)

BCS=breast conserving surgery; RT=radiotherapy.

Number of cases excluded from each variable owing to unknown status is as follows: stage; 1175 cases, surgery allocation; 47 cases, radiotherapy; 197 cases, hormone therapy; 107 cases, chemotherapy; 196 cases, appointment delay; 4048 cases, treatment delay; 382 cases.

a BCS before mastectomy.

b Proportion of patients that received RT, by type of surgery.

**Table 2:** Association between socioeconomic status and patient and treatment factors (Odds Ratios per quartile increase in Townsend score)

	**Unadjusted OR (95% CI)**	**Age-adjusted OR (95% CI)**	**Age-and stage-adjusted OR (95% CI)**
Age <64 years	1.13 (1.10–1.16)		
Stage III/IV	1.14 (1.10–1.19)	1.13 (1.08–1.18)	
Surgical treatment	0.82 (0.79–0.85)	0.83 (0.80–0.87)	0.85 (0.80–0.91)
Breast conserving surgery^a^	0.89 (0.87–0.92)	0.92 (0.89–0.95)	0.92 (0.89–0.95)
Mastectomy^a^	1.09 (1.06–1.12)	1.09 (1.06–1.12)	1.08 (1.05–1.12)
Radiotherapy	0.88 (0.86–0.91)	0.90 (0.88–0.93)	0.91 (0.88–0.94)
BCS and radiotherapy^b^	0.95 (0.90–1.02)	0.96 (0.89–1.02)	0.97 (0.90–1.03)
Chemotherapy	0.96 (0.93–0.99)	1.01 (0.97–1.04)	0.97 (0.93–1.01)
Hormone therapy	1.04 (1.00–1.08)	1.02 (0.99–1.06)	1.04 (1.00–1.08)
Appointment delay >14 days	1.06 (1.02–1.09)	1.06 (1.03–1.10)	1.07 (1.03–1.11)
Treatment delay >14 days	1.02 (1.00–1.05)	1.03 (1.00–1.06)	1.05 (1.02–1.08)

BCS=breast-conserving surgery; CI=confidence interval; OR=odds ratio.

a Analysis includes surgery patients only.

b Analysis includes BCS patients only.

**Table 3:** Association between socioeconomic status and 5-year survival from breast cancer for all patients and by type of surgery

	**Unadjusted HR (95% CI)**	**Age-adjusted HR (95% CI)**	**Age-and stage adjusted HR (95% CI)**
All patients	1.14 (1.11–1.16)	1.11 (1.08–1.13)	1.08 (1.05–1.11)
Breast-conserving surgery group	1.13 (1.07–1.19)	1.14 (1.08–1.20)	1.13 (1.07–1.19)
Mastectomy group	1.05 (1.01–1.09)	1.04 (1.00–1.08)	1.04 (1.00–1.08)
No surgery group	1.04 (1.00–1.08)	1.04 (1.00–1.08)	1.03 (0.98–1.08)

CI=confidence interval; HR=hazard ratio.
